# NMR and LCMS analytical platforms exhibited the nephroprotective effect of *Clinacanthus nutans* in cisplatin-induced nephrotoxicity in the in vitro condition

**DOI:** 10.1186/s12906-020-03067-3

**Published:** 2020-10-22

**Authors:** Ilya Iryani Mahmod, Intan Safinar Ismail, Noorjahan Banu Alitheen, Yahaya M. Normi, Faridah Abas, Alfi Khatib, Rudiyanto, Jalifah Latip

**Affiliations:** 1 Laboratory of Natural Products, Institute of Bioscience, Universiti Putra Malaysia, 43400 Serdang, Selangor Malaysia; 2 Department of Chemistry, Faculty of Science, Universiti Putra Malaysia, 43400 Serdang, Selangor Malaysia; 3 Department of Cell and Molecular Biology, Faculty of Biotechnology and Biomolecular Sciences, Universiti Putra Malaysia, 43400 Serdang, Selangor Malaysia; 4 Department of Food Science, Faculty of Food Science and Technology, Universiti Putra Malaysia, 43400 Serdang, Selangor Malaysia; 5 Faculty of Pharmacy, International Islamic University Malaysia, 25200 Kuantan, Pahang Malaysia; 6 Faculty of Fisheries and Food Science, Universiti Malaysia Terengganu, 21030, Kuala Nerus, Terengganu Malaysia; 7 School of Chemical Science and Food Technology, Faculty of Science and Technology, Universiti Kebangsaan Malaysia, 43600 Bandar Baru Bangi, Selangor Malaysia

**Keywords:** Nephroprotective effect, *Clinacanthus nutans*, NRK-52E, Cisplatin

## Abstract

**Background:**

*Clinacanthus nutans* (*C. nutans*) Lind. locally known as Belalai Gajah or Sabah snake grass is a medicinal plant belonging to Acanthaceae family. In Asia, this plant is traditionally used for treating skin rashes, insects and snake bites, diabetes mellitus, fever and for diuretic effect. *C. nutans* has been reported to possess biological activities including anti-oxidant, anti-inflammation, anti-cancer, anti-diabetic and anti-viral activities.

**Methods:**

Proton Nuclear Magnetic Resonance (^1^H NMR) and Liquid Chromatography Mass Spectroscopy (LCMS) coupled with multivariate data analysis were employed to characterize the metabolic variations of intracellular metabolites and the compositional changes of the corresponding culture media in rat renal proximal tubular cells (NRK-52E).

**Results:**

NMR and LCMS analysis highlighted choline, creatine, phosphocholine, valine, acetic acid, phenylalanine, leucine, glutamic acid, threonine, uridine and proline as the main metabolites which differentiated the cisplatin-induced group of NRK-52E from control cells extract. The corresponding media exhibited lactic acid, glutamine, glutamic acid and glucose-1-phosphate as the varied metabolites. The altered pathways perturbed by cisplatin nephrotoxic on NRK-52E cells included changes in amino acid metabolism, lipid metabolism and glycolysis.

**Conclusion:**

The *C. nutans* aqueous extract (1000 μg/mL) exhibited the most potential nephroprotective effect against cisplatin toxicity on NRK-52E cell lines at 89% of viability. The protective effect could be seen through the changes of the metabolites such as choline, alanine and valine in the *C. nutans* pre-treated samples with those of the cisplatin-induced group.

**Supplementary information:**

**Supplementary information** accompanies this paper at 10.1186/s12906-020-03067-3.

## Background

Cisplatin (*cis*-Diamminedichloroplatinum (II)) is an important chemotherapeutic agent which is useful in the treatment of several cancers [1]. Unfortunately, there are major side effects in using this chemotherapeutic agent, whereby clinical nephrotoxicity or acute kidney injury occurs in about 30% of the patients [2]. Clinically, cisplatin nephrotoxicity is often seen after 10 days of cisplatin administration, which resulted in a lower glomerular filtration rate, and higher serum creatinine and reduced serum magnesium and potassium levels [3]. On the other hand, exposure of tubular cells to cisplatin leads to cell injury and even cell death. A high concentration of cisplatin induced necrotic cell death in confluent monolayers of proximal tubule cells, whereas lower concentrations led to apoptosis [4]. Kidney damage caused by cisplatin is found in proximal tubular S3 portion, the distal tubule and collecting duct. The cisplatin nephrotoxicity is due to the possibility that it generates the reactive metabolites which covalently bound to cellular molecules [5]. The binding of the platinum (II) to protein bound sulfhydryl (thiol) group (−SH) is supposed to cause the cisplatin nephrotoxicity. The detection of reduction in sulfhydryl groups in the rat renal cortex has been demonstrated to occur before any significant changes in renal function which suggesting that this change might be a crucial event. In cell fractionations, the highest decline of sulfhydryl groups occurs in the mitochondrial and cytosol fractions having the highest concentrations of platinum [6]. Therefore, nephrotoxicity via cisplatin induction on proximal tubule kidney cells was seen to be worthwhile in observing the possible outcome by medicinal plants as a method of looking for alternative nephroprotective agents. Many medicinal plants have been proven to be as nephroprotective agents but there is still lack of any scientific evidence to support such claims. Nephroprotective agents are the elements which possess protective effect against nephrotoxicity. Medicinal plants have curative properties because of the presence of numerous complex chemical substances [7]. The nephroprotective activity of various medicinal plants can be administered by the presence of the nephrotoxic agent to induce nephrotoxicity in the model. Medicinal plants possess nephroprotective properties due to the presence of various bioactive principles such as alkaloids, flavonoids, naphthoquinone, saponins, tannins and triterpenes, which assist in decreasing the rate of nephrotoxicity [8, 9]. Among the medicinal plants that are consumed for their nephroprotective effects included *Rubia cordifolia* Linn. (root), *Boerhaavia diffusa* (root), *Aerva javanica* (fresh roots), *Curcuma longa* (rhizome), *Ficus religiosa* L. (latex), *Tectona grandis* (bark), *Strychnos potatorum* (seed), *Carica papaya* (seed), *Crataeva nurvala* (fruit), *Tamarindus indica* (fruit pulp), *Punica granatum* L. (fruit peel), *Euphorbia neriifolia* (leaf), *Vernonia cinerea* (aerial part), *Acorus calamus* (aerial part), *Aerva lanata* (whole plant), and *Orthosiphon stamineus* (whole plant) [10].

The need for dialysis and adverse effect of dialysis can be reduced by the use of medicinal plants that help in treating the causes and effects of renal failure [11]. Many studies have revealed that, clinically useful drugs, like cisplatin, acetaminophen and gentamicin have many adverse effect and can cause organ toxicities through the metabolic activation to highly reactive free radicals which includes superoxide and reactive oxygen species [12, 13]. Therefore, it is worthwhile to evaluate the potential plants which could be useful as nephroprotective agents, which might help to reduce or minimize the nephrotoxicity of drugs like cisplatin, gentamicin and acetominophen. A study has demonstrated that carotenoids, like lycopene, are very strong antioxidants and can prevent the body from the toxic effects of other substances [14]. Carotenoids can prevent cell damage due to ROS by react chemically with reactive oxygen species (ROS) and oxidize [15]. The findings suggested that the use of lycopene, caused decrease in serum BUN, Cr, and other measured biomarkers after cisplatin administration. Thus, lycopene administration significantly resulted to protect kidneys against the cisplatin-induced nephrotoxicity [15]. The oxidative agents play an important role in a kidney tissue damage and cisplatin-induced nephrotoxicity [16]. Thus, the role of antioxidants from plants should be further investigated in looking for treatment of kidney damage.

*Clinacanthus nutans* (*C. nutans*) Lind. or locally known as Belalai Gajah from Acanthaceae family, is a small shrub native to tropical Asia [17]. In Malaysia, the fresh leaves of the plant are usually boiled in water and consumed as herbal tea. In Thailand, alcoholic extract of fresh leaves is used externally for treatment of skin rashes, snake and insect bite, herpes simplex virus and varicella-zoster virus (VZV) lesions. This plant is also used traditionally for diabetes mellitus, fever and diuretic. *C. nutans* has been reported to possess anti-oxidant [18, 19], anti-inflammation [20] and anti-viral activity [21, 22]. Previous phytochemical studies showed various identified bioactive compounds from this plant including flavonoids, sulfur-containing glycosides [23], diglycerides [24], chlorophyll [25], cerebrosides and a monoacylgalactosylglycerol [26]. Despite all the known biological activities from the previous works, emerging lay testimonies and Malaysian newspaper reports advocated that *C. nutans* might possess nephroprotective effect. However, these testimonies were not supported by any scientific evidence. Thus, this study was molded to evaluate the nephroprotective effects of *C. nutans* on in vivo rat kidney cell line (NRK-52E) by NMR and LCMS metabolomics approach.

## Methods

### Plant materials and extraction

*Clinacanthus nutans* plants were collected in Sendayan, Negeri Sembilan (GPS coordinates: 2.77^o^ N, 101.99^o^ E) in June 2014 and identified by a botanist, Dr. Shamsul Khamis, from Institute of Bioscience (IBS), Universiti Putra Malaysia. The plant voucher specimen (Acquisition no. SK 2883/15) was deposited at the herbarium of the Laboratory of Natural Products, IBS. The leaves were dried under shady condition at room temperature (25–30 °C) for 7 days. The leaves were then ground in a blender to a fine powder. Subsequently, extractions using different ratios of ethanol to water (0 to 100%, with 20% increment) were performed by sonication for 30 min at 30–40 °C. The extraction steps were repeated three times and the extracts were pooled before being evaporated under pressure at 40 °C to yield the crude leaf extracts.

### Drug and plant extract preparation for cell assay

Cisplatin from Sigma-Aldrich (St. Louis, USA) was prepared into a stock solution in the concentration of 3.33 mM before the treatment on the cells was performed with 5, 10, 20, 30 and 40 μM of cisplatin respectively. Due to the non-solubility of some of the extracts obtained from *C. nutans*, they were dissolved in media containing DMSO (20 μL DMSO in 180 μL media) and the concentration of DMSO administered to cells was maintained below 0.2% at the highest concentration of the extract in the well.

### Cell culture

Rat renal proximal tubular cells (NRK-52E) were obtained from American Type Culture Collection (ATCC CRL-1571, USA), and were frozen in liquid nitrogen before cultivation. Cells were passaged every 2–3 days in 75 cm^3^ culture flask containing Dulbecco’s modified medium supplemented with 10% fetal bovine serum, 0.1 mM non-essential amino acids, 4 mM L-glutamine, 100 U/mL penicillin and 100 μg/mL streptomycin (Invitrogen). The cells were maintained at 37 °C in a 5% CO_2_ humidified atmosphere. Cells were sub-cultured or harvested for experiments when reached about 90% confluency. For experimental purposes, cells were used between passages 5 and 15.

### Cell viability assay

#### MTT assay

The MTT assay was conducted in accordance to the method described by Mosmann (1983) [27] with slight modifications. NRK-52E cells were plated in a 96-well plate 24 h before the treatment at the density of 5.0 × 10^4^ per well. The cells were then incubated in 37 °C CO_2_ incubator overnight. In the following day, NRK-52E cells were pretreated with seven different extracts of *C. nutans* at different concentrations (1000, 500, 250, 125, 62.3, and 31.1 μg/ml) and incubated for another 24 h. Induction with cisplatin at the concentration of 20 μM was done for the next 48 h. The normal cells without any treatment were used as control. Cell viability was measured after 72 h of treatment. MTT solution (5 mg/ml) was added at a volume 20 μl into each well and incubated for 3 h. The solution was discarded before DMSO was added to dissolve the dark blue crystal. The viability of the treated cells were recorded at 570 nm using a microplate reader (SPECTRAmax PLUS, Sunnyvale, CA, USA). Each reported point represents the mean of triplicate well in three independent experiment.

#### Lactate dehydrogenase (LDH) assay

The LDH assay was conducted according to the manufacturer’s (Promega, USA) protocol. Cells were cultured overnight in 96-well plate at the density of 5.0 × 10^4^ cells/well. The cells were pretreated with *C. nutans* extracts for 24 h before inducing with cisplatin (20 μM) for another 48 h. The normal cells without any treatment were used as control. The CytoTox-ONE reagent and stop solution were then added before measuring the fluorescence produced due to excitation at 530–570 nm and emission at 580–620 nm using fluorescence microplate reader (SPECTRAmax PLUS, Sunnyvale, CA, USA).

### Sample preparation for NMR analysis

For the purpose of analysing cells’ metabolites with and without treatment of *C. nutans* extracts, culture media was firstly removed from the cell culture flasks. The cells obtained were washed twice with cold phosphate buffer saline (PBS, pH 7.4). The cells were then quenched with cold HPLC-grade methanol (Merck, Darmstadt, Germany) and collected by detaching them from the culture dish using a cell scrapper. The normal cells without any treatment with *C. nutans* extract and cisplatin were used as control.

The metabolites in the cultured NRK-52E cells were extracted using water extraction method adopted from Matheus (2014) [28] with slight modification. Briefly, 300 μL of D_2_O containing phosphate buffer (pH 7.4) was added to each 1.5 mL centrifuge tube containing the dried quenched cells. The sample was then subjected to ultrasonication for 15 min and centrifuged at 1000 g for 10 min. The obtained supernatant was transferred into another sample tube. The extraction process was repeated for each sample. Finally, 20 μL of trimethylsilylpropanoic acid (TSP) solution (2.5 mg/mL) was added to 250 μL of each sample and the mixed well mixture was transferred to a 3 mm NMR tube. For samples involving the respective culture media, 50 μL D_2_O (containing 0.1% TSP) was added to 550 μL of the media sample. The sample mixture was vortexed and transferred to a 5 mm NMR tube.

#### NMR analysis of cell extract and corresponding culture media

NMR spectra of all cell extracts and their corresponding culture media were recorded at 298 K on Bruker Ascend 700 MHz spectrometer equipped with a cryogenic probe. ^1^H NMR spectrum of each sample was acquired using one dimensional Nuclear Over Hauser Effect Spectroscopy (NOESY). The NOESY experiment was chosen due to its better sensitivity and more efficiency in water suppression compared to water presaturation experiment. The acquisition time for each ^1^H NMR spectrum was 3.53 min, consisting of 64 scans with a width of 12 ppm. Additional support for identification was obtained using two-dimensional (2D) NMR such as J-resolved (JRES), homonuclear spectroscopy (COSY) and heteronuclear multiple bond coherence (HMBC).

#### NMR data processing and multivariate data analysis

All raw ^1^H NMR spectra of cell extract and its corresponding culture media were manually phased, baseline corrected and referenced to the TSP resonance at 0.00 ppm using Chenomx NMR suite (Chenomx NMR Suite 5.1 Professional, Edmonton, Canada). Each spectrum was segmented into bins with a width of 0.04 between chemical shift regions of 0.0 to 10.0 ppm. The bins of the residual water (4.52–4.92) were excluded from all the NMR spectra. The output data in Microsoft Excel were exported to SIMCA-P 13.0 software package (Umetrics, Umeå, Sweden) for statistical analysis.

The metabolite identification of cell and culture media was achieved by comparison with Chenomx NMR suite 7.7 (Chenomx Inc., Edmonton, Canada), Human Metabolite Database (HMDB, http://www.hmdb.ca/), and reported data in literature.

### Sample preparation for LCMS analysis

Cell lysates and their corresponding media were diluted ten times with HPLC-grade methanol. The diluted sample was transferred to a 1.5 mL vial.

#### Q-TOF UPLC-MS analysis

UPLC analysis was performed on an Acquity HILIC column (1.8 μm, 2.1 × 100 mm; Waters) with the column temperature maintained at 45 °C. Methanol was used as the mobile phase in an isocratic system with constant flow rate set at 0.3 mL min^− 1^. The injection volume was 10 μL per sample. MS detection was carried out using Q-TOF UPLC/MS (Agilent Technologies, USA), operated in both positive and negative ion modes. The capillary voltages were 3.5 kV (ESI+) and 2.5 kV (ESI-), cone voltages were at 35 V (ESI+) and 30 V (ESI-). Cone gas flow was set at 50 L h^− 1^ with the source temperature at 120 °C. Desolvation gas flow was maintained at 500 L h^− 1^ with the desolvation gas temperature at 300 °C. Data were collected in the centroidmode. The mass range was set at *m/z* 80–1000 with a scan time of 0.20 s and inter-scan time of 0.02 s. For MS/MS analysis, the collision energy was set in a ramp mode ranging from 10 V to 40 V.

#### Q-TOF UPLC-MS data processing and analysis

All the spectra obtained were evaluated using RStudio software. The software was employed for alignment, framing and metabolite identification through HMDB database by using Metabolite Automatic Identification Toolkit (MAIT). The peak alignment and framing were done based on *m/z* and retention time intervals of 0.002 and 0.1 min, respectively. The RStudio software extracted the raw UPLC-MS data and produced a matrix of retention time – mass electric charge ratio (Rt-*m/z*) variables pair with *m/z* peak intensity data for each sample. The list of compounds identified across the selected database was based on the accurate mass matching. The PCA was applied to visualise the data after scaled in unit variance (UV). The OPLS-DA model was then used to determine the peaks that significantly contributed to the separation and to further classify the treatment effect. The S-plot was used to explain the possible correlation and in identifying the statistical discriminants between two groups. All data analyses were executed by SIMCA-P+ version 12 (Umetrics AB, Umeå, Sweden).

### Statistical analysis

The pathway analysis was done using Metaboanalyst 3.0 (http://www.metaboanalyst.ca) [29]. Univariate analysis was performed by using the integration area data of each metabolite. One-way analysis of variance (ANOVA) and t-test was done using GraphPad Prism V 7.0 (GraphPad Software Inc., San Diego, CA, USA). Tukey’s test was chosen as the post-hoc analysis method in which *p* ≤ 0.05 was considered statistically significant, and the values were expressed as mean ± SEM.

## Results

### MTT assay for *C. nutans* extracts on rat renal proximal tubular cells (NRK-52E)

**Fig. 1:**
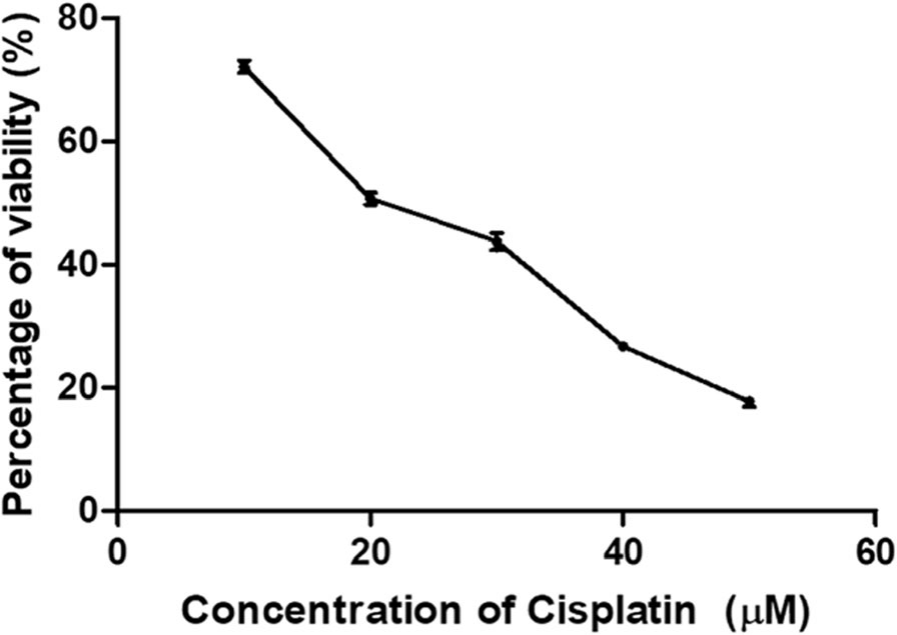
The percentage viability of NRK-52E treated with different concentrations of cisplatin for 48 h

Previous study has reported that cisplatin concentrations from 10 to 50 μM may lead to the NRK-52E cells apoptosis [30]. Hence, the present study used cisplatin concentrations at 10, 20, 30, 40 and 50 μM to evaluate its toxicity on the NRK-52E cell line. The obtained results showed that cell proliferation decreased in the dose and time-dependent manner. The cell viability decreased to 51.3% in the group treated with 20 μM for 48 h implying half lethal concentration (LC_50_) (Fig. 1). Hence, the cisplatin dose of 20 μM for the induction in 48 h was used in the MTT assay.

**Fig. 2:**
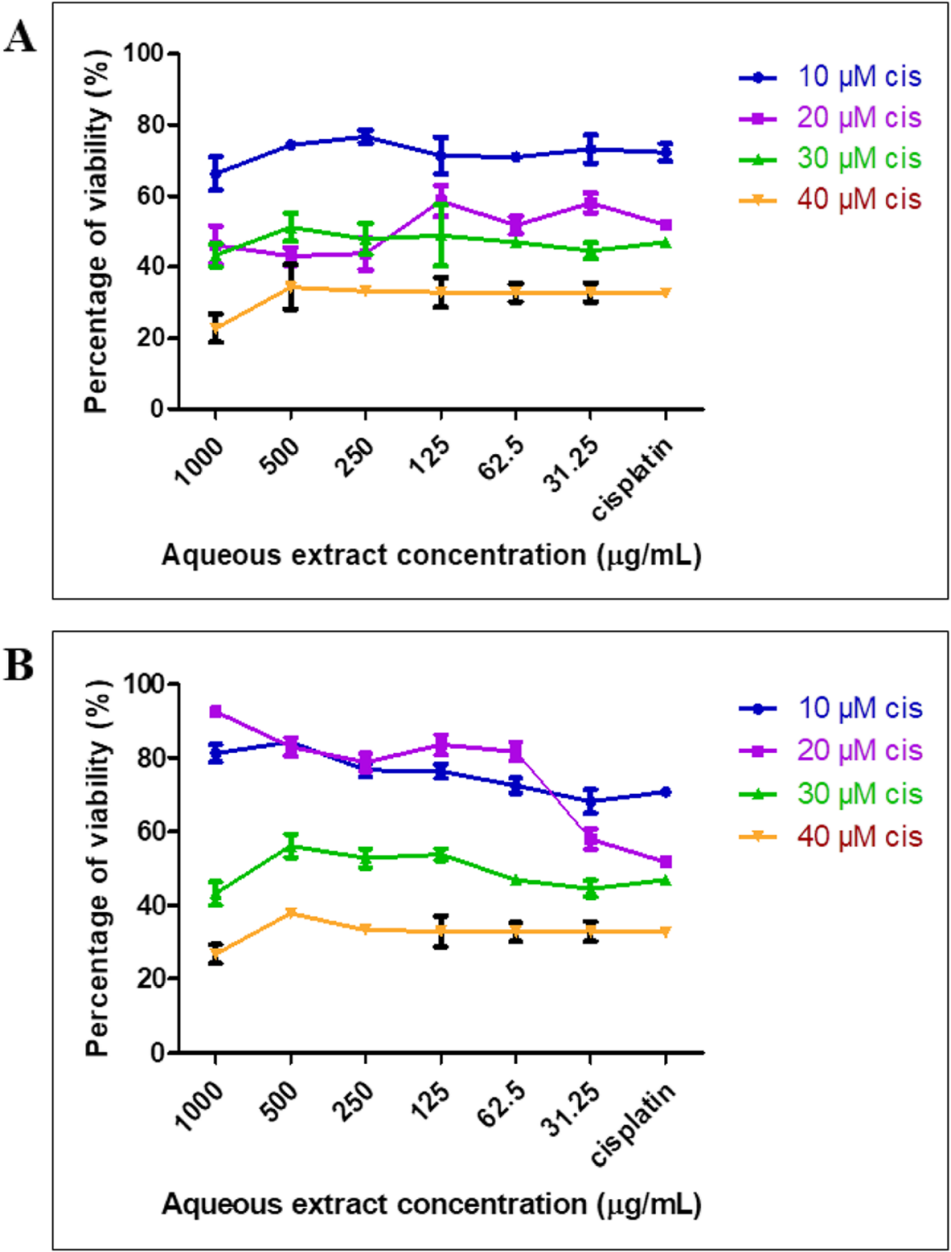
Percentage viability of NRK-52E by pre-treatment of aqueous extract (μg/ml) at different concentrations with immediate induction (**a**) and 24 h before induction (**b**) until 48 h of cisplatin at different concentrations

MTT assay was conducted to assess the possible nephroprotective effect of six *C. nutans* extracts (0, 20, 40, 60, 80, 100% ethanol in water) on NRK-52E cells. The nephroprotective condition was defined as an improvement in the percentage viability of the cells when pre-treated with the extracts. The aqueous extract of *C. nutans* exhibited the most nephroprotective effect against the cisplatin toxicity with 89% percentage viability compared to treatment with cisplatin alone (51.3%). However, the protective effect was only observed when the cells were pre-treated (prophylaxis) with the *C. nutans* aqueous extract, but not in a post-treatment method (Additional file 1: Figure A1). NRK-52E cells pre-treated with different concentrations (1000, 500, 250, 125, 62.5 and 31.3 μg/ml) of the aqueous extract followed by immediate exposure to 20 μM cisplatin for 48 h did not protect against cisplatin induced-apoptosis (Fig. 2a). Conversely, when a certain period was introduced between the pre-treatment of extract and cisplatin exposure, a protective effect could be observed. The effect was seen after 24 h of the extract pre-treatment followed by 48 h of cisplatin exposure (Fig. 2b). Based on the observations as shown in Fig. 2a and b, the parameter of the 24 h pre-treatment period and 48 h of 20 μM cisplatin exposure was used in the subsequent experiments.

**Fig. 3:**
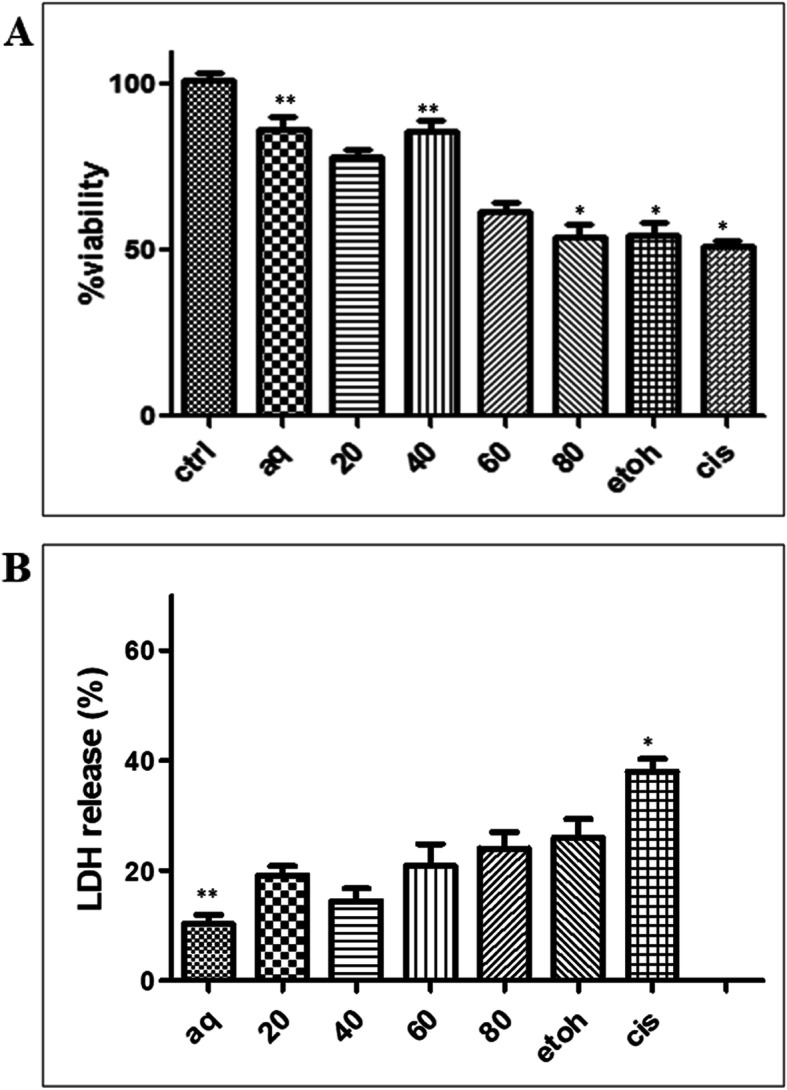
**a** MTT assay on NRK-52E after pre-treated with different solvent extracts of *C. nutans* at 1000 μg/ml for 24 h followed by 48 h treatment with cisplatin at 20 μM. **b** LDH analysis on NRK-52E after pre-treated with different *C. nutans* extracts at 1000 μg/ml for 24 h followed by 48 h induction with cisplatin at 20 μM. (20–20% ethanol extract; 40–40% ethanol extract; 60–60% ethanol extract; 80–80% ethanol extract; EtOH-100% ethanol extract and cis-20 μM cisplatin). The relative LDH release (%) related to control wells containing cell culture medium without any treatment. *Statistically significant difference compared to control (*p* < 0.05). ** Statistically significant difference compared with cisplatin (*p* < 0.05)

Figure 3a shows that the aqueous extract of *C. nutans* leaf exhibited the highest protective effect with 89% viability on the cisplatin-induced cell compared to the cisplatin group (51.3% viability) without any pre-treatment with CN extract. The LDH assay was also conducted to analyse the protective effect of the extract towards cisplatin induction at 20 μM (Fig. 3b). These results are in agreement with the results of the plant extract on the NRK-52E that have been shown in the MTT assay (Fig. 3a).

### ^1^H NMR analysis of NRK-52E cell extract and corresponding culture media

**Fig. 4:**
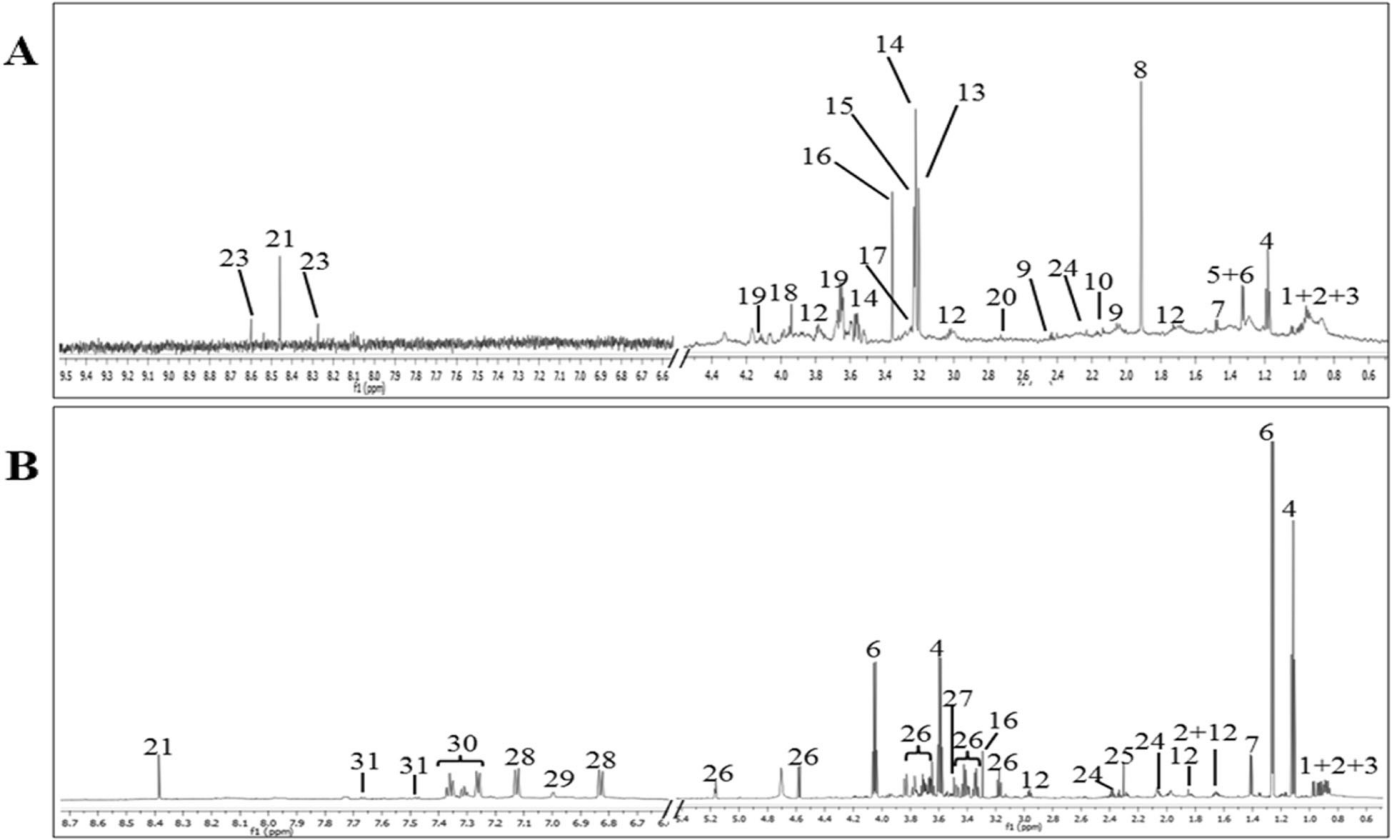
Identified metabolites from cell extract (**a**) and culture media (**b**). -(1) isoleucine, (2) leucine, (3) valine, (4) ethanol, (5)threonine,(6) lactate, (7) alanine, (8) acetate, (9) glutamate, (10) glutathione, (11) aspartate, (12) lysine, (13) phosphocholine, (14) choline, (15) glycerophosphocholine, (16)scyllo-inositol, (17) betaine, (18) creatine, (19)myo-inositol, (20) dimethylamine, (21) formate, (22)glucose-1-phosphate, (23)ATP,ADP,AMP, (24) glutamine, (25) pyruvate, (26) glucose, (27) glycine, (28) tyrosine, (29) histidine, (30) phenylalanine, (31) tryptophan (32)cholate. *Aromatic region (5.8–11.0 ppm) in (A) is at 3 times vertical expansion*

^1^H NMR spectral representatives of aqueous cell extract and culture media of NRK-52E are shown in Fig. 4a and b**.** All of the metabolites were identified (Table 1) according to the literature data [31–33] and the Human Metabolome Database (www.hmdb.ca) followed by confirmation with 2D NMR (J-Resolved).

**Table 1:** Chemical constituents identified in the cell extract

Peak	Metabolites	δ_**H**_ ppm (multiplicity)
**1**	Isoleucine	0.95 (d, 7.4 Hz), 1.00 (d, 7.0 Hz)
**2**	Leucine	0.96 (d, 6.5 Hz)
**3**	Valine	0.99 (d, 6.8 Hz), 1.05 (d, 6.8 Hz)
**4**	Ethanol	1.35 (d)
**5**	Threonine	1.33(d, 6.5 Hz), 4.24 (m)
**6**	Lactate	1.33(d, 6.7 Hz), 4.11 (m)
**7**	Alanine	1.48 (d, 7.2 Hz)
**8**	Acetate	1.95 (s)
**9**	Glutamate	2.06 (m), 2.12 (m), 2.35 (m)
**10**	Glutathione	2.17 (m), 3.78 (m)
**11**	Aspartate	4.59 (d, 8.0 Hz), 2.83 (m)
**12**	Lysine	4.73 (d, 8.0 Hz), 4.10 (m), 2.94 (s)
**13**	Phosphocholine	4.74 (m), 3.47 (s)
**14**	Choline	3.24 (s)
**15**	Glycerophosphocholine	4.74 (m), 3.47 (s)
**16**	Scyllo-inositol	3.34 (s)
**17**	Betaine	3.89 (s)
**18**	Creatine	3.04 (s), 3.94 (s)
**19**	Myo-inositol	4.06 (t,10 Hz), 3.55 (dd, 2.9, 3.0 Hz), 3.63 (t, 9.5 Hz)
**20**	Dimethylamine	2.73 (s)
**21**	Formate	8.45 (s)
**22**	Glucose-1-phosphate	5.67d
**23**	ATP,ADP,AMP	7.84 (m), 8.63(m)
**24**	Glutamine	2.14 (m), 3.77 (m)
**25**	Pyruvate	2.35 (s)
**26**	Glucose	5.22 (d, 3.8 Hz)
**27**	Glycine	3.56 (s)
**28**	Tyrosine	3.42 (t, 5.1 Hz)
**29**	Histidine	6.98 (m)
**30**	Phenylalanine	7.32 (m), 7.37 (m)
**31**	Tryptophan	7.67 (m)

*J* coupling values (in Hz). Multiplicity: singlet (s), doublet (d), triplet (t), doublet of doublet (dd), multiplet (m)

The NMR spectra of the cell extract showed the domination by alanine (δ1.48), acetate (δ1.95), glutamate (δ2.06, δ2.13, δ2.44), leucine (δ0.96), valine (δ0.99, δ1.05), lactate (δ1.33, δ4.24), phosphocholine (δ4.74, δ3.47), choline (δ3.24), glycerophosphocholine (δ4.74, δ3.47), among others. In the ^1^H NMR spectra of corresponding culture media, the metabolites such as threonine (δ1.33, δ3.56), isoleucine (δ1.02, δ1.46, δ8.09), valine (δ0.99, δ1.05, δ2.28), alanine (δ1.48, δ3.78), and glutamine (δ2.13, δ2.44) were identified. Multivariate data analysis was further applied to obtain a more detailed analysis of the metabolic differences between different treatment groups.

### Metabolic variation of NRK-52E cells and culture media due to cisplatin nephrotoxic

**Fig. 5:**
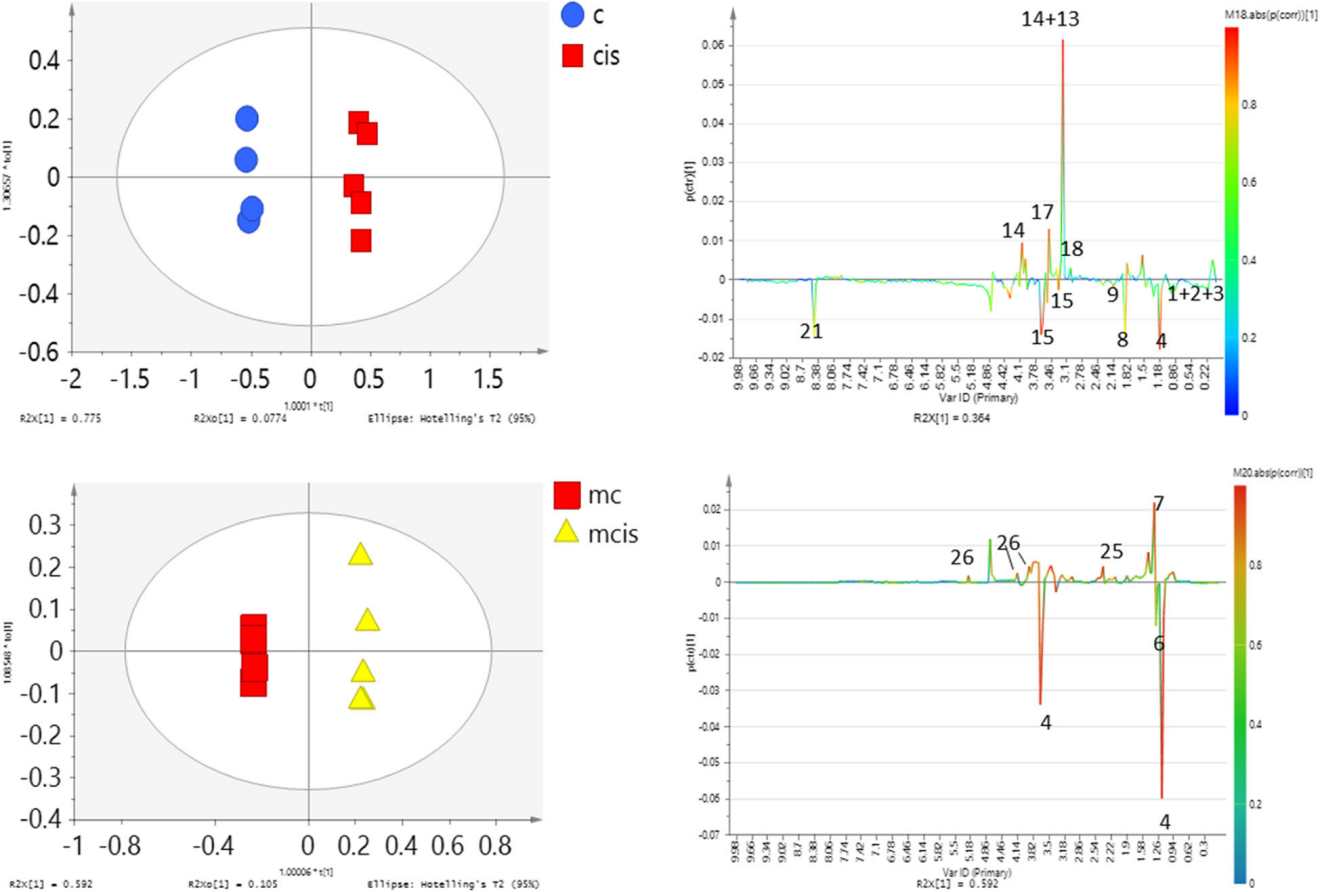
OPLS-DA scores derived from ^1^H NMR spectra of NRK-52E cell extracts and culture media from control (C and mc) and cisplatin-treated groups (CIS and mcis) and corresponding coefficient plots

The NMR profile between the control and treated group of NRK-52E cells extract and culture media exhibited obvious metabolite differences. Based on the supervised orthogonal projection to latent structures-discriminant analysis (OPLS-DA) score plot in Fig. 5, higher levels of choline, creatine, and phosphocholine were detected in the cisplatin group. However, valine, lysine, and leucine were more concentrated in the control group. These suggested that cisplatin could have induced alteration in the metabolism of NRK-52E cells. The permutation test for the explained variation (R2 = 0.91) and predictive capability (Q2 = 0.85) were significantly high, indicating the satisfactory predictive ability of the model (Additional file 2: Figure A2).

According to the variable importance parameter (VIP) results (Additional file 3: Figure A3), 10 of 23 variables had greater variability (VIP value > 1.0) indicating that these metabolites play important roles in the changes due to the treatments on NRK-52E. The significant metabolites that responsible for the separation between control and cisplatin group were shown in S-line plots (Fig. 5a). The contribution of the metabolites was presented by colours from blue to red, red being the highest while blue being the lowest impact. In Fig. 5a, the upper plot of the loading plot represented metabolites increased in the cisplatin treatment group for the cell extract: choline, phosphocholine, creatine, lysine, and creatine. Whereas the section below represented metabolites increased in the control group: leucine, ethanol, alanine, glutamate, betaine and glycine. Figure 5b depicts the metabolites that are different between control and cisplatin induced group from the culture media. The relative quantification of these putative biomarkers was carried out and their fold change values are shown in Tables 2 and 3.

**Table 2:** Major metabolite changes observed in normal: control (C) and cisplatin-induced (CIS) of NRK-52E cells extract, ***p < 0.0001, **p < 0.001, *p < 0.05

No.	Metabolite	*δ*_H_ ppm (multiplicity)	Fold changes	Metabolic pathway
1	Acetate	1.90 (s)	0.42**	Glycolysis
2	Alanine	1.46 (d, 7.2 Hz)	0.70**	Amino acid metabolism
3	Choline	3.18 (s)	4.07***	Lipid metabolism
4	Creatine	3.02 (s)	1.66***	Energy metabolism
5	Ethanol	1.17(t)	0.48**	Glycolysis
6	Glutamate	2.14 (m), 2.38 (m)	0.20*	Amino acid metabolism
7	Glycerophosphocholine (GPC)	3.22 (s)	0.44**	Lipid metabolism
8	Lactate	1.30 (d, 6.7 Hz)	0.42**	Glycolysis
9	Lysine	1.54 (m)	1.62***	Amino acid metabolism
10	Phosphocholine	3.20 (s), 3.58 (m), 4.15 (m)	1.33***	Lipid metabolism

**Table 3:** Major metabolite changes observed in normal control (MC) and cisplatin-induced of NRK-52E culture media

No.	Metabolite	*δ*_H_ ppm (multiplicity)	Fold changes	Metabolic pathway
1	Alanine	1.48 (d)	1.10***	Amino acid metabolism
2	Ethanol	1.17 (t), 3.65 (dd)	0.57**	Glycolysis
3	Glucose	5.22 (d)	1.05***	Glycolysis
4	Lactate	1.30 (d)	0.93**	Glycolysis
5	Pyruvate	3.36 (s)	1.14***	Glycolysis

***p < 0.0001, **p < 0.001

### Selection of biomarkers by LCMS profile (NRK-52E)

More than 400 peaks of metabolites in the LCMS spectrum for each sample of cell extract were assigned using the HMDB database through the MAIT processor. Peaks from the spectra that were missing in more than 10% of the sample from each treatment group were omitted from further analysis. The use of the database resulted in a total of approximately 220 identified metabolites which were found common in all groups.

**Fig. 6:**
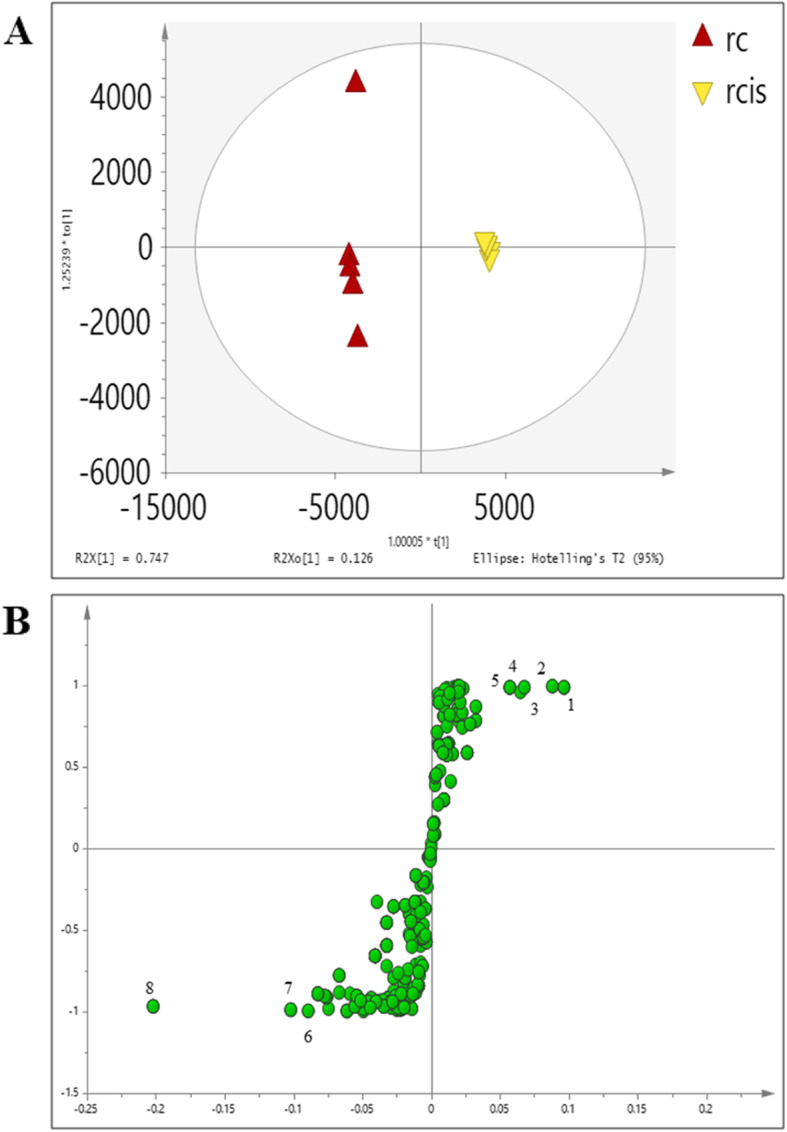
The OPLSDA score (**a**) and S plots (**b**) of control vs a cisplatin-induced group of the cell extract. Metabolites; (1) glutamic acid, (2)L-threonine, (3) uridine, (4)L-proline, (5)L-leucine, (6)L- valine, (7)L-serine, (8)L-phenylalanine,(9)L-glutamine

In this study, the OPLS-DA was applied to reveal a clearer observation on the significant metabolites that related to the separation between control (**rc**) and cisplatin-induced (**rcis**) cells (Fig. 6a). There was an obvious separation between **rc** and **rcis**, with good discriminant statistical values of R2 and Q2 of 95.6 and 92.8, respectively. In the OPLS-DA model, a CV-ANOVA value < 0.05 was required for the model to be considered valid [34] . The CV-ANOVA value was 3.39e-007 indicating the optimum fit and hence reconfirmed the validity of this model (Additional file 4: Figure A4).

The VIP was used to rank the selected variables based on their contribution to the model. The ranked list was then generated, following the selection of the compounds at each end of the S-plot to separate the up- and down-regulated compounds. The S-plot is the covariance and correlation loading diagnostics of the OPLS-DA model. This model shows an overview of the affecting variables and filters the important metabolites in the projection. The significantly increased metabolites in the model are in the upper-right quadrant of the S-plot, which have positive correlations and co-variances as shown in Figs. 6b (cell extract) and 7b (media).

**Fig. 7:**
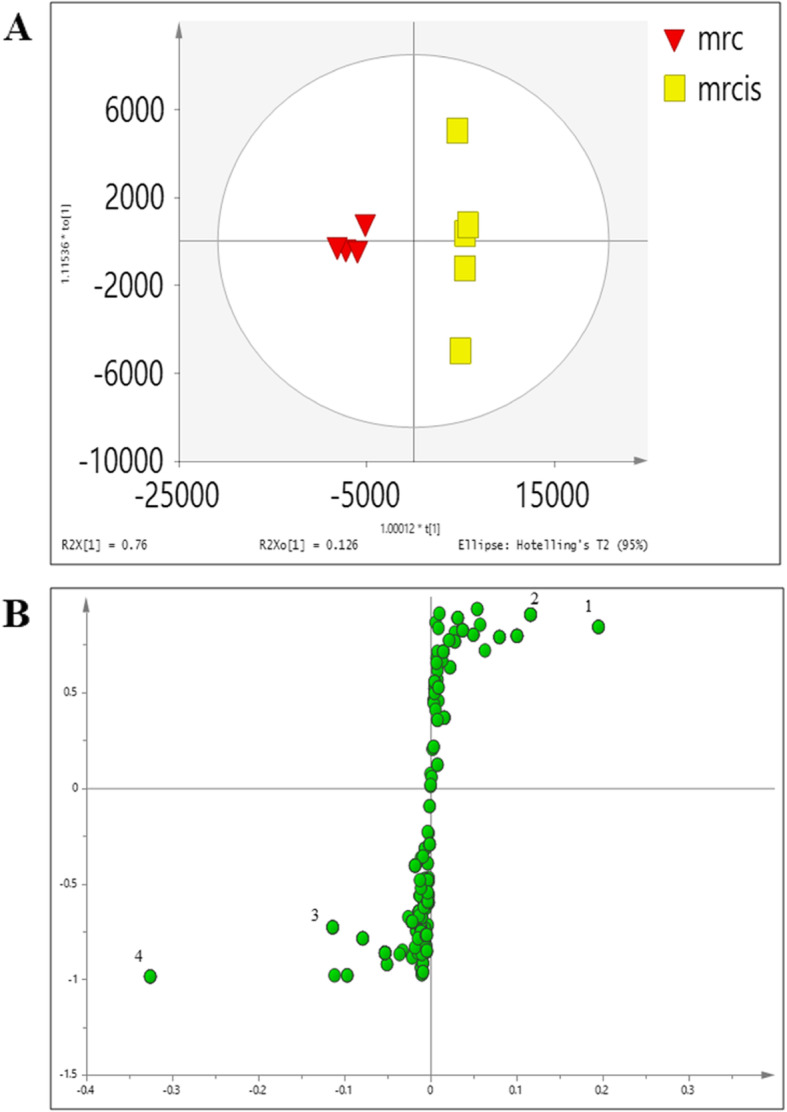
The OPLSDA score (**a**) and S plots (**b**) of control vs a cisplatin-induced group of the corresponding media. Metabolites; (1) lactic acid, (2) glutamic acid, (3) glucose 1-phosphate, and (4)glutamine

The biomarker identification was then carried out by using MAIT (Metabolite Identification Toolkit). MAIT is capable of peak detection for metabolomics LC/MS data sets. It uses a matched filter [35] and the centWave algorithm [36] through the XCMS package, developed by the SCRIPPS Centre for Metabolomics (San Diego, US). This package holds a metabolite identification stage to search for the significant masses by using a tolerance window according to the database of Human Metabolome Database (HMDB) [37].

Table 4 shows the list of putative metabolites from LC-MS along with their masses, retention times and their metabolic pathways. The results suggested 9 identified metabolites from cell extract which were significantly affected by cisplatin exposure. The metabolites are valine, acetic acid, phenylalanine, leucine, glutamic acid, threonine, uric acid, and proline. The affected metabolites in the corresponding media are as listed in Table 5 wherein some metabolites namely glutamine and glutamic acid were similar to the ones detected in the cell extract.

**Table 4:** Identified metabolite from cell extract of NRK-52E based on library annotation

Compound	RT (min)	m/z	Mass error (ppm)	Fold change	Metabolic pathway
**Glutamic acid**	1.3772	146.0458	2.92	1.03	Amino acid metabolism
**Threonine**	1.4594	118.0505	1.09	4.03	Amino acid metabolism
**Uridine**	1.3753	243.0622	2.54	2.45	Amino acid metabolism
**L-Proline**	1.5592	114.0558	2.61	1.21	Amino acid metabolism
**L-Leucine**	0.9015	130.0871	2.67	1.19	Amino acid metabolism
**L-Valine**	1.4134	116.0714	1.96	0.75	Amino acid metabolism
**L-Serine**	0.6656	104.0349	1.24	0.55	Amino acid metabolism
**L-Phenylalanine**	1.4391	164.0708	2.24	0.49	Amino acid metabolism
**L-Glutamine**	0.9056	145.0617	3.22	0.31	Amino acid metabolism

**Table 5:** Identified metabolite from corresponding culture media of NRK-52E based on library annotation

Compound	RT (min)	m/z	Mass error (ppm)	Fold change	Metabolic pathway
Lactic acid	1.2749	89.0719	3.33	1.87	Glycolysis
Glutamic acid	1.1733	146.0458	2.92	1.55	Amino acid metabolism
Glucose 1-phosphate	1.1952	259.0211	3.05	0.74	Glycolysis
L-Glutamine	1.2735	145.0617	3.22	0.46	Amino acid metabolism

The identification of the metabolites derived from the NMR and LC-MS analyses showed differences in several significant metabolites in the control and cisplatin-induced group. However, the combination NMR and LCMS methods resulted in metabolic profiles that provided access to higher number of identified metabolites. The predictive power of the model in both sensitivity and specificity using these analytical platforms was better when compared with the results from a single analytical technique [38].

To understand the metabolic changes in NRK-52E and its corresponding culture media which could lead to an understanding of the mechanism involved, the cells were pre-treated with the *C. nutans* aqueous extract (1000 μg/ml) for 24 h followed with exposure to cisplatin (20 μM) for another 48 h. The cells and culture media were then harvested after 48 h of the cisplatin exposure.

**Fig. 8:**
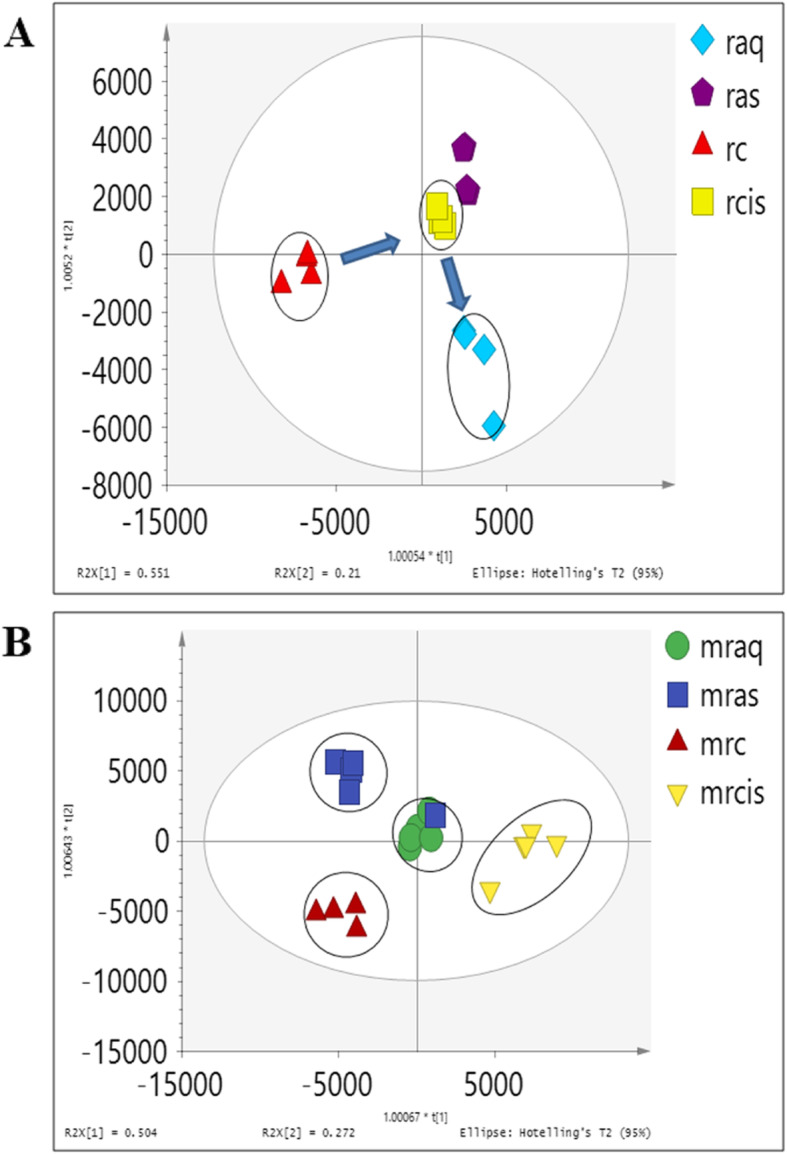
The OPLSDA score (**a**) cell extract and (**b**) corresponding media showing the trajectories of different treatment on NRK-52E. c: control. Cis: cisplatin induction without any pre-treatment. aq: cisplatin induction with pre-treatment of *C. nutans* aqueous extract. as: pre-treatment with aqueous extract without any induction with cisplatin

The OPLS-DA score plot in Fig. 8a shows the pre-treatment of aqueous extract on the cell extract (raq) separated from the cisplatin (rcis) induced group. The secretome of the treated group could be seen approaching the control group as shown in Fig. 8b suggesting that treatment with aqueous extract of *C. nutans* has experienced some changes in the metabolites of NRK-52E in response to cisplatin exposure. Figure 8b of OPLS-DA scores derived from ^1^H NMR spectra of NRK-52E culture media extracts from control (mc) and cisplatin-treated groups (mcis) and corresponding coefficient plots. The major metabolites which showed significant changes are as listed in Tables 6 and 7.

**Table 6:** Major metabolite changes observed in normal: control (C), cisplatin induced (CIS) and aqueous extract pre-treatment (AQ) of NRK-52E cells extract, ***p < 0.0001, **p < 0.001, *p < 0.05

Metabolite	***δ***_**H**_ ppm (multiplicity)	CIS vs C	AQ vs C
**Acetate**	1.90 (s)	0.42**	0.51**
**Alanine**	1.46 (d, 7.2 Hz)	0.70**	1.16***
**Choline**	3.18 (s)	4.07***	2.26***
**Creatine**	3.02 (s)	1.66***	1.76***
**Ethanol**	1.17(t)	0.48**	0.61**
**Glutamate**	2.14 (m), 2.38 (m)	0.20*	0.43**
**Glycerophosphocholine (GPC)**	3.22 (s)	0.44**	0.35*
**Lactate**	1.30 (d, 6.7 Hz)	0.42**	0.92**
**Lysine**	1.54 (m)	1.62***	0.74**
**Phosphocholine**	3.20 (s), 3.58 (m), 4.15 (m)	1.33***	1.61***

**Table 7:** Major metabolite changes observed in normal control (MC), cisplatin induced (MCIS) and aqueous extract pre-treatment (MAQ) of NRK-52E culture media

Metabolite	***δ***_**H**_ ppm (multiplicity)	CIS	AQCN + CIS
**Alanine**	1.48 (d)	1.10***	1.56***
**Ethanol**	1.17 (t), 3.65 (dd)	0.57**	1.29***
**Glucose**	5.22 (d)	1.05***	1.01***
**Lactate**	1.30 (d)	0.93**	1.11***
**Pyruvate**	3.36 (s)	1.14***	1.26***

***p < 0.0001, **p < 0.001

In NMR analysis, the effect of *C. nutans* aqueous extract against cisplatin on NRK-52E was quantitatively determined relative to the concentration of the internal reference standard, trimethylsilyl propionic acid (TSP). The concentration of the metabolites in NRK-52E cells extract and culture media of treated and non-treated groups and their relative changes were expressed in fold change value compared to control (control = 1). Metabolites included choline, phosphocholine, lactate, acetate, formate, which are known to be involved in multiple metabolic processes (lipid metabolism and amino acids metabolism) were among those who experienced detectable changes.

Multivariate data analysis was employed to analyse the metabolite variation due to the different treatments on NRK-52E cells. To determine the metabolic response of NRK-52E cells due to the presence of the *C. nutans* extracts followed by induction of cisplatin, the OPLS-DA model was performed. As shown in Fig. 8a, good separation in the score plot by PC1 was obtained between the control group and the cisplatin-induced group. The permutation test for the explained variation (R2 = 0.91) and predictive capability (Q2 = 0.85) was significantly high, indicating the satisfactory validity of the model (Additional file 4, Figure A4).

**Fig. 9:**
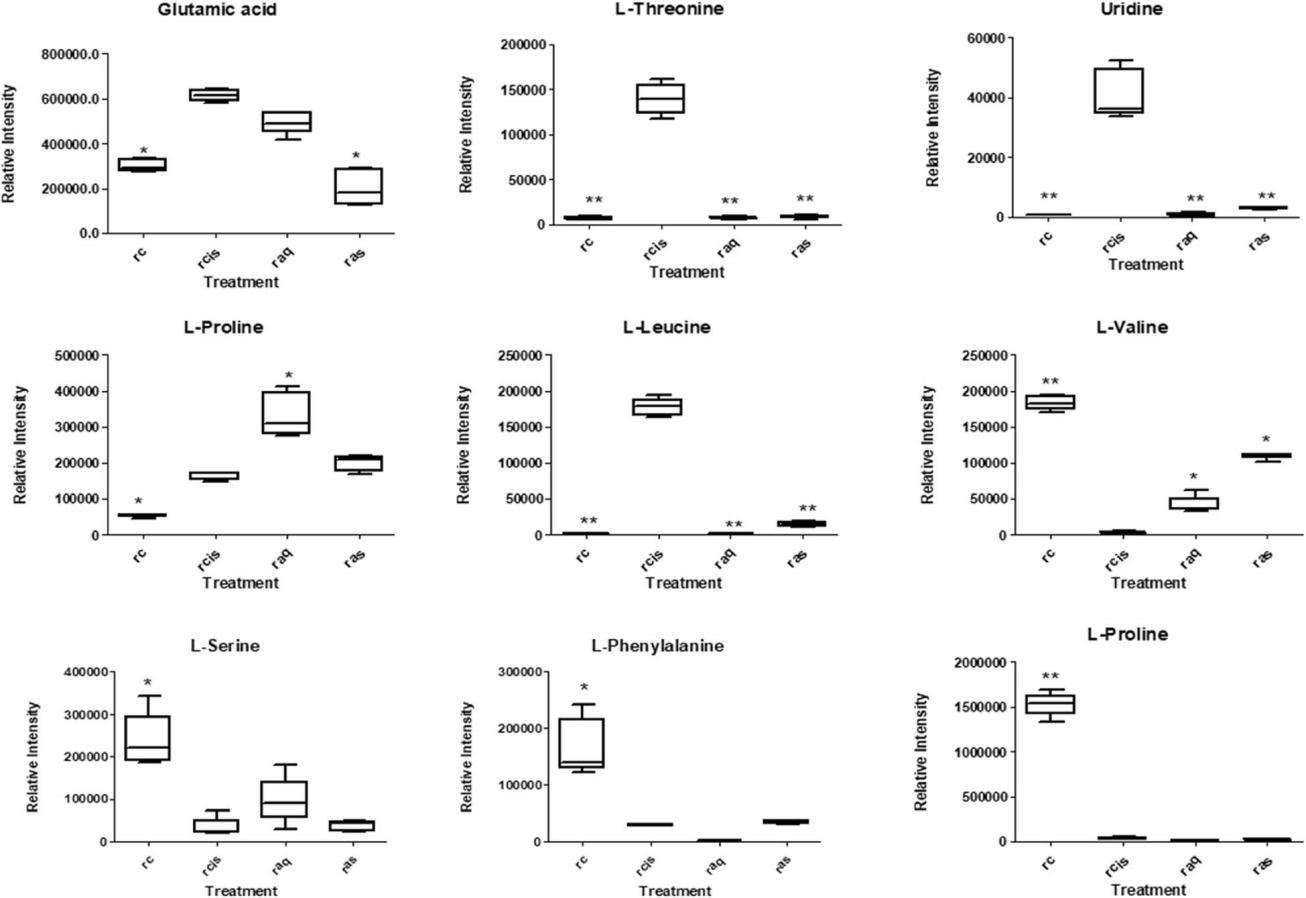
The box and whisker plots of the relative quantities of the most significant metabolites in the cell extract samples from the treated and untreated groups. Normal (rc), cisplatin-induced (rcis), aqueous pre-treatment (raq), and pre-treatment with aqueous extract without any induction with cisplatin (ras) group. Intensity of metabolites (expressed as MEAN ± SEM) when comparing treatment groups and the cisplatin-induced group. ANOVA of significant value of T-test, *p* < 0.05, * and *p* < 0.01, **

**Fig. 10:**
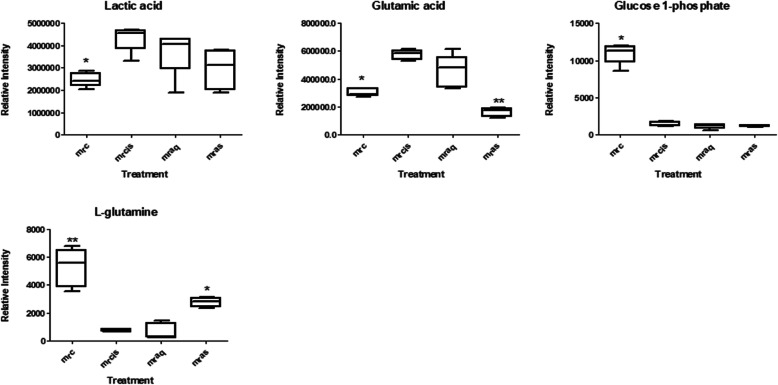
The box and whisker plots of the relative quantities of the most significant metabolites in the corresponding media samples from the treated and untreated groups. Normal (mrc), cisplatin-induced (mrcis), aqueous pre-treatment (mraq), and pre-treatment with aqueous extract without any induction with cisplatin (mras) group. Intensity of metabolites (expressed as MEAN ± SEM) when comparing treatment groups and the cisplatin-induced group. ANOVA of significant value of T-test, *p* < 0.05, * and *p* < 0.01, **

Levels of the metabolites between the treatment groups were compared in Tables 2 and 3 in fold change for NMR data and using the box-and-whisker plot for LC-MS data (Figs. 9 and 10). The trajectory in the score plot obtained of LCMS data (Fig. 8a and b) displays a clear shift of *C. nutans* aqueous extract (aq) treatment to the bottom right quadrant after induction with cisplatin, suggesting that this group has changed due to the induction (cis). The changes caused by aqueous *C. nutans* leaf extract (aq) pre-treatment on the levels of the metabolite biomarkers identified in cisplatin nephrotoxicity were visualized in the box whisker plots of Figs. 9 and 10.

## Discussion

### Proposed perturbed pathways involved in nephrotoxic of cisplatin by NMR and LCMS analysis

The present in vitro study demonstrated the nephroprotective potential of *C. nutans* aqueous extract in cisplatin induced nephrotoxicity by NMR and LCMS approach. Numerous experimental studies showed that cisplatin causes nephrotoxicity [5, 39–41]. Cisplatin-induced nephrotoxicity occurs due to production of reactive oxygen species (ROS), especially hydroxyl radicals, which leads to lipids peroxidation, oxidation of proteins, lipids, nucleic acids, and cell membrane [42].

**Fig. 11:**
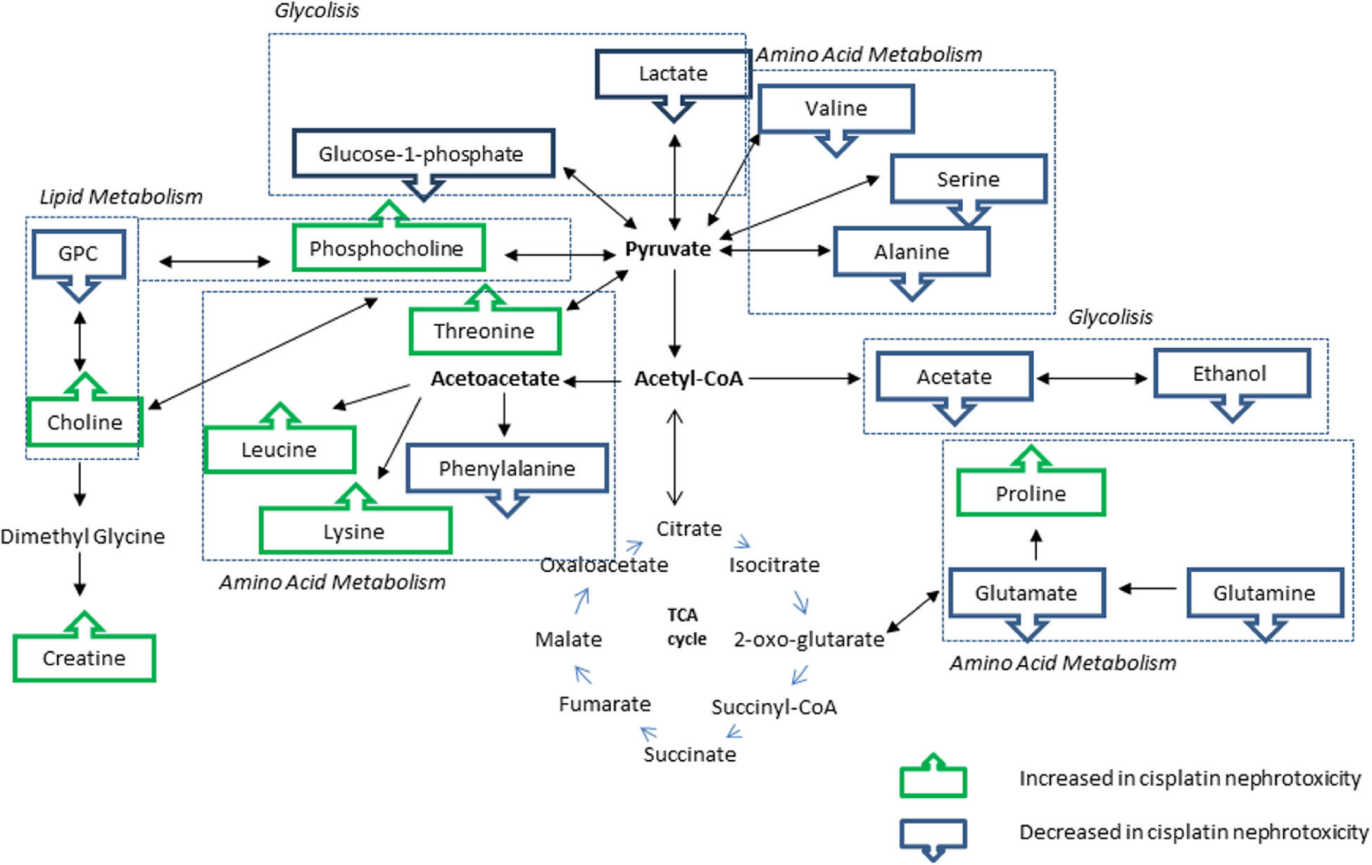
Altered metabolic pathways for the metabolite differences in cells between cisplatin-induced nephrotoxic compared to the control group

The identified metabolite perturbation, based on the data of ^1^H NMR and LCMS cell extract and the corresponding culture media between the nephrotoxic and normal condition, suggested that specific metabolic pathway alterations have occurred. Identification of the metabolic pathways associated with specific metabolite changes could improve the understanding on the biological condition of normal trajected to the nephrotoxic condition in cells. The altered pathways by cisplatin and/or *C. nutans* extract include purine metabolism, amino acid metabolism, and glycolysis metabolism, which were further analysed using MetaboAnalyst, KEGG, and HMDB based on the identified key metabolites or biomarkers. These databases are comprehensive for high-throughput metabolomics data analysis to reveal the most relevant pathway which was affected by cisplatin and/or the *C. nutans* extracts. Figure 11 shows a detail pathway map with metabolite markers identified using both NMR and LC-MS methods for cisplatin nephrotoxicity in NRK-52E cells.

#### Phospholipid metabolism

The effect of cisplatin exposure could be seen clearly on choline and choline-containing compounds, phosphocholine (PC) and glycerophosphocholine (GPC). The intracellular level of choline was observed to be increased after the cells were exposed to cisplatin induction with 4.07 folds compared to control (Table 2). Levels of choline metabolites are well known to be altered in the states of hyperproliferation [31]. Choline and its derivatives are important constituents in phospholipid metabolism of the cell membrane and were identified as markers in cell proliferation [33]. A major decrease was observed in the GPC level of cisplatin-induced groups suggesting the increase of membrane degradation since GPC degraded into free choline [43] which contributed to the intense choline peak in these two treatments compared to the control. This might cause changes in phospholipids biosynthesis rather than an increase in choline accumulation from culture media since the media used in these studies did not show choline. Previous studies have reported that a decreased level of renal osmolytes like GPC was observed in renal papilla necrosis [44].

#### Amino acid metabolism

The most significant responses altered by the different treatments on the cells were the decrease in many amino acid levels. Amino acids that showed significant decreases were alanine, valine, serine, phenylalanine, and glutamine in the cisplatin-induced groups. The involvement of amino acids in nephrotoxicity was reported earlier and alanine has been used as a potential biomarker for end-stage renal cortical toxicity in several studies [44–46]. In this study, alanine along with several amino acids were observed to be significantly altered, suggesting that the nephrotoxicity on cells was successfully being induced by cisplatin at the selected concentration.

#### Glycolysis

The ethanol level in the intracellular was observed to be decreased in the cisplatin nephrotoxic group. Ethanol is a production from the anaerobic cell respiration process [47]. In the cisplatin exposed group, the decrease in ethanol might indicate the reduction in alcohol dehydrogenase activity in the glycolysis process [48].

The reduction of glucose uptake from media into the cell could be observed in the cisplatin-induced group as the level of the glucose increased in the cells compared to the control group. This reduction might inhibit the rate of glycolysis. In addition to glucose consumption, the extracellular pyruvate, lactate, and alanine were also observed. There was a major decrease in the lactate level in the culture media of the cisplatin treatment group without an increase in glucose uptake over the same period. This might be due to the enhancement of mitochondrial metabolism. Lactate and acetate release are closely correlated with the variance of glucose utilization due to the main cytosolic pyruvate production from glycolysis [32].

### Nephroprotective effect of *C. nutans*

The treatment with CN aqueous extract has reduced the level of choline in 2 folds compared to a cisplatin-induced group, suggesting that the membrane degradation occurred due to cisplatin has been reduced. Increased level of alanine (Table 6) and valine (Fig. 9) could be observed in the CN pre-treatment group which suggesting disturbance in amino acid metabolism. From this study, cisplatin nephrotoxic has caused a decrease in amino acids (alanine, valine, serine, phenylalanine, and glutamine). Xu et al., (2008) [46] has reported alanine being used as a potential biomarker for end-stage renal cortical toxicity. Previous study has shown that the amino acid-like proline has been reduced in HK-2 cells when the cells were induced by cisplatin [49]. This occurrence might be due to the utilization of amino acids to fuel up the energy in treating the injured cells via the TCA cycle [50]. Hence, it could be summarized that the CN aqueous pre-treatment on NRK-52E cells has improved the cell viability due to toxicity caused by cisplatin through the altering of the lipid and amino acid pathways.

Previous studies showed that most medicinal plants are rich in anti-inflammatory compounds and antioxidants wherein the plants nephroprotective effects could be related to these compounds [8, 10, 51]. The nephroprotective effect of CN in the present study could be related to the presence of anti-inflammatory properties and antioxidant. The study by Khoo et al., 2018 [52], suggested that sulphur-containing glucosides, sulphur containing compounds, phytosterols, triterpenoids, flavones and some organic and amino acids in the air-dried water leaf extract to be the main contributors to the anti-inflammatory properties of CN. Thiol or sulphur containing compounds have been reported to be clinically used to reduce nephrotoxicity of cisplatin [5]. High doses of reduced glutathione injected intravenously within 30 min of cisplatin administration rendered a protective sign in kidney [53]. Another study on cisplatin induced nephrotoxicity suggested the nephroprotective effect of garlic on rat was due to the antioxidant property and thiol rich compounds [54].

From this study, it could be proven that the medicinal plants may have potential roles against nephrotoxicity. A variety of medicinal plants have been reported for their significant in vitro and in vivo nephroprotective activity. The results of this study indicated that aqueous extract of CN leaves have potential for use against kidney damage particularly in the proximal tubular cell membrane. Hence, a further study is needed to establish the more detailed mechanism of nephroprotective activity of the aqueous CN leaves extract.

## Conclusions

Metabolic profiling of cell extract and corresponding culture media of NRK-52E undertaken in this study using NMR and LC-MS analysis, along with multivariate data analysis methods have given a detailed picture of metabolic changes in the cells and media due to different treatments of the *Clinacanthus nutans* aqueous extract compared to the control group. A number of significant metabolites changes were detected between the groups are particularly noteworthy since these metabolites, varied gradually from the control group, might potentially be useful biomarkers in detecting the nephrotoxicity and nephroprotection in in vitro cells and media. Hence, this study has successfully paved a preliminary assay in screening for potential alternative nephroprotective agents from the plants.

## Supplementary information

**Additional file 1.** MTT assay result for post treatment method for all the extract.

**Additional file 2.** Permutation test for OPLS-DA scores derived from 1H NMR spectra of NRK-52E cell extracts.

**Additional file 3.** 10 important variable VIP value greater than 1.

**Additional file 4.** Permutation test for OPLSDA score and S plots of control vs a cisplatin-induced group of the cell extract.

## Data Availability

The datasets used and analysed during the current study are available from the corresponding author on reasonable request.

## Abbreviations

S3: Third segment
VZV: Vricella-zoster virus
NRK-52E: Rat kidney cell line
NMR: Nuclear magnetic resonance
LCMS: Liquid chromatography mass spectrometry
DMSO: Dimethyl sulfoxide
ATCC: AMERICAN Type Culture Collection
MTT: 3-(4,5-dimethylthiazol-2-yl)-2,5-diphenyl tetrazolium bromide
LDH: Lactate dehydrogenase
PBS: Phosphate buffer saline
HPLC: High performance liquid chromatography
D_2_O: Deuterium oxide
TSP: Trimethylsilyl propionic acid
NOESY: Nuclear Over Hauser Effect Spectroscopy
JRES: J-resolved spectroscopy
COSY: Homonuclear spectroscopy
HMBC: Heteronuclear multiple bond coherence
Q-TOF UPLC-MS: Quadrupole-time of flight ultra-performance liquid chromatography mass-spectrometry
MS: Mass spectrometry
MS/MS: Tandem mass spectrometry
ESI: Electrospray ionization
HMDB: Human Metabolome Database
MAIT: Metabolite Automatic Identification Toolkit
PCA: Principle component analysis
OPLS-DA: Orthogonal projection to latent structures-discriminant analysis
