# Supplementary material for: NMR and LCMS analytical platforms exhibited the nephroprotective effect of Clinacanthus nutans in cisplatin-induced nephrotoxicity in the in vitro condition
Source: BMC Complement Med Ther. 2020 Oct 22;20:320. doi: 10.1186/s12906-020-03067-3 (PMC7579835; doi:10.1186/s12906-020-03067-3)
Supplement: Supplementary file 1 — Additional file 1. MTT assay result for post treatment method for all the extract. [file 12906_2020_3067_MOESM1_ESM.docx]

**Additional file 1.** MTT assay result for post treatment method for all the extract

|  |  | **Cell Viability(%)** | |  |  |  |
| --- | --- | --- | --- | --- | --- | --- |
| **Type of plant extract** |  | **Extract Concentration (µg/mL)** | | |  |  |
|  | **1000** | **500** | **250** | **125** | **62.5** | **31.25** |
| **CN (100 % EtOH)** | 36.22±0.17 | 62.20±0.48 | 64.68±0.69 | 62.48±1.03 | 60.42±0.99 | 52.40±0.45 |
| **CN (80 % EtOH)** | 33.87±1.12 | 59.76±1.98 | 60.76±0.87 | 60.02±3.02 | 60.76±2.98 | 57.98±1.67 |
| **CN (60 % EtOH)** | 41.88±2.01 | 50.76±1.32 | 55.98±1.44 | 59.98±1.59 | 60.87±0.95 | 57.98±1.77 |
| **CN (40 % EtOH)** | 66.70±0.86 | 68.20±0.76 | 61.37±0.64 | 62.52±0.11 | 60.23±0.73 | 51.46±0.46 |
| **CN (20 % EtOH)** | 59.07±1.97 | 61.73±1.11 | 68.15±1.76 | 66.08±0.78 | 68.31±1.03 | 51.06±1.65 |
| **CN (Aqueous)** | 69.62±1.94 | 63.70±1.33 | 65.44±2.13 | 61.74±0.99 | 63.91±1.76 | 50.46±1.35 |
