# Supplementary material for: NMR and LCMS analytical platforms exhibited the nephroprotective effect of Clinacanthus nutans in cisplatin-induced nephrotoxicity in the in vitro condition
Source: BMC Complement Med Ther. 2020 Oct 22;20:320. doi: 10.1186/s12906-020-03067-3 (PMC7579835; doi:10.1186/s12906-020-03067-3)
Supplement: Supplementary file 2 — Additional file 2. Permutation test for OPLS-DA scores derived from 1H NMR spectra of NRK-52E cell extracts. [file 12906_2020_3067_MOESM2_ESM.docx]

**Additional file 2** Permutation test for OPLS-DA scores (**H)** derived from ^1^H NMR spectra of NRK-52E cell extracts
