# Supplementary material for: NMR and LCMS analytical platforms exhibited the nephroprotective effect of Clinacanthus nutans in cisplatin-induced nephrotoxicity in the in vitro condition
Source: BMC Complement Med Ther. 2020 Oct 22;20:320. doi: 10.1186/s12906-020-03067-3 (PMC7579835; doi:10.1186/s12906-020-03067-3)
Supplement: Supplementary file 3 — Additional file 3. 10 important variable VIP value greater than 1. [file 12906_2020_3067_MOESM3_ESM.docx]

**Additional file 3.** 10 important variable VIP value greater than 1

| **Var ID (Primary)** | **M1.VIP[1]** | **1.89456 * M1.VIP[1]cvSE** |
| --- | --- | --- |
| **3.18** | 1.28467 | 0.303847 |
| **1.88** | 1.12778 | 0.229617 |
| **2.12** | 1.17483 | 0.891824 |
| **1.18** | 1.13261 | 0.315295 |
| **1.44** | 1.11742 | 0.178574 |
| **1.28** | 1.08134 | 0.188362 |
| **3.2** | 1.06361 | 0.374647 |
| **1.56** | 1.06022 | 0.217722 |
| **1.32** | 1.04807 | 0.217155 |
| **3.265** | 1.02972 | 0.382228 |
