# Supplementary material for: NMR and LCMS analytical platforms exhibited the nephroprotective effect of Clinacanthus nutans in cisplatin-induced nephrotoxicity in the in vitro condition
Source: BMC Complement Med Ther. 2020 Oct 22;20:320. doi: 10.1186/s12906-020-03067-3 (PMC7579835; doi:10.1186/s12906-020-03067-3)
Supplement: Supplementary file 4 — Additional file 4. Permutation test for OPLSDA score and S plots of control vs a cisplatin-induced group of the cell extract. [file 12906_2020_3067_MOESM4_ESM.docx]

**Additional file 4.** Permutation test for OPLSDA score (**J**) and S plots (**K**) of control vs a cisplatin-induced group of the cell extract
